# Decentralized Computation Offloading Strategy via Multi-Agent Deep Reinforcement Learning for Multi-Access Edge Computing Systems

**DOI:** 10.3390/s26030914

**Published:** 2026-01-30

**Authors:** Emmanuella Adu, Yeongmuk Lee, Jihwan Moon, Sooyoung Jang, Inkyu Bang, Taehoon Kim

**Affiliations:** 1 IDEACONCERT Co., Ltd., Seongnam 13449, Republic of Korea; 2 Department of Computer Engineering, Hanbat National University, Daejeon 34158, Republic of Korea; 3 Department of Mobile Convergence Engineering, Hanbat National University, Daejeon 34158, Republic of Korea; 4 Department of Intelligence Media Engineering, Hanbat National University, Daejeon 34158, Republic of Korea

**Keywords:** multi-access edge computing, offloading, grant-free access, deep reinforcement learning, task completion latency

## Abstract

Multi-access edge computing (MEC) has been widely recognized as a promising solution for alleviating the computational burden on edge devices, particularly in supporting fast and real-time processing of resource-intensive applications. In this paper, we propose a decentralized offloading decision strategy based on multi-agent deep reinforcement learning (MADRL), aiming to minimize the overall task completion latency experienced by edge devices. Our proposed scheme adopts a grant-free access mechanism during the initialization of offloading in a fully decentralized manner, which serves as the key feature of our strategy. As a result, determining the optimal offloading factor becomes significantly more challenging due to the simultaneous access attempts from multiple edge devices. To resolve this problem, we consider a discrete action space-based deep reinforcement learning (DRL) approach, termed deep Q network (DQN), to enable each edge device to learn a decentralized computation offloading policy based solely on its local observation without requiring global network information. In our design, each edge device dynamically adjusts its offloading factor according to its observed channel state and the number of active users, thereby balancing local and remote computation loads adaptively. Furthermore, the proposed MADRL-based framework jointly accounts for user association and offloading decision optimization to mitigate access collisions and computation bottlenecks in a multi-user environment. We perform extensive computer simulations using MATLAB R2023b to evaluate the performance of the proposed strategy, focusing on the task completion latency under various system configurations. The numerical results demonstrate that our proposed strategy effectively reduces the overall task completion latency and achieves faster convergence of learning performance compared with conventional schemes, confirming the efficiency and scalability of the proposed decentralized approach.

## 1. Introduction

The recent proliferation of computationally intensive applications, such as virtual reality (VR), augmented reality (AR), autonomous driving, and real-time online gaming, has significantly increased the processing demands on mobile and Internet of Things (IoT) devices [1]. These emerging applications require not only powerful computing capabilities but also ultra-low latency and high energy efficiency for real-time operation. However, conventional IoT and mobile devices are constrained by limited processing power and battery capacity, which makes executing such complex tasks locally highly challenging [2]. Accordingly, multi-access edge computing (MEC) has been introduced as a key enabler to bridge this gap by offloading computational tasks from resource-constrained devices to nearby edge servers located at the network edge [3]. By enabling computation within radio access proximity, MEC reduces transmission delay and core-network congestion while allowing distributed intelligence and context awareness. Consequently, MEC has been considered an essential component in next-generation wireless networks, particularly in 6G systems aiming to support ubiquitous connectivity and massive device access [4,5].

Nevertheless, efficient offloading decision-making remains a crucial challenge in MEC. Each device must determine whether to execute its task locally or offload it partially or fully to the MEC server. Most of the previous studies focus on minimizing task-completion latency or energy consumption at the users or MEC servers [6], whereas some also focus on jointly optimizing both. To minimize the average task completion latency of users, the authors in [7] employed a block coordinate descent (BCD) method to jointly optimize offloading decision, computation, and bandwidth allocation resources during partial offloading. Tun et al. also optimized the task offloading decision, resource allocation, and trajectory of unmanned aerial vehicles (UAVs) to jointly minimize the energy consumption and task execution latency in MEC-equipped UAV networks [8]. Akhavan et al. proposed a deep reinforcement learning-based offloading decision and resource management (DECENT) algorithm to optimize the offloading decision and computing resource allocation of each arriving task in real time [9]. Although these algorithms aid in either achieving a better computation experience or energy efficiency, most of them considered a centralized algorithm for either a single-user or multi-user scenario. However, these centralized algorithms lead to high system overhead and considerable latency, as the base station (BS) is required to collect global information from all the users in the environment before making a decision and also distributing the decision made back to the users [10].

Recent studies on the MEC and computation offloading have progressively evolved from simple offloading decision problems toward more comprehensive formulations that jointly consider resource constraints, virtualization overhead, latency requirements, energy consumption, mobility, and communication limitations. Early works investigated dynamic resource allocation mechanisms in NFV-enabled MEC systems to support ultra-reliable and low-latency communications (URLLC), where computation offloading was modeled as a stochastic and dynamic system aiming to simultaneously satisfy service delay constraints and system stability, rather than merely maximizing throughput [11]. Subsequently, research efforts addressed resource-constrained MEC scenarios with unreliable or limited communication conditions, proposing distributed and cooperative offloading structures that enable terminal devices to make effective offloading decisions without centralized control, thereby redefining offloading in realistic MEC environments [12]. In large-scale IoT settings, the limitations of conventional TDMA and NOMA schemes motivated the design of novel NOMA-based computation offloading and device scheduling frameworks, in which offloading decisions are tightly coupled with multiple access mechanisms to improve overall system throughput [13]. This line of research was further extended to NOMA-aided MEC systems, where task offloading and computing resource allocation were jointly optimized to minimize energy consumption under stochastic task arrivals and time-varying wireless channels, without relying on prior statistical knowledge [14]. Beyond terrestrial networks, MEC research has expanded toward aerial and space platforms, including UAV-assisted and STAR-RIS-enabled MEC systems, where computation offloading, communication resources, and mobility are jointly optimized under dynamic line-of-sight conditions and energy constraints [15]. In satellite MEC scenarios, particularly those involving low Earth orbit (LEO) constellations, collaborative and distributed offloading schemes have been proposed to cope with spatio-temporal variability and intermittent connectivity, formulating offloading as a cooperative decision-making problem among satellites [16]. Hierarchical aerial MEC architectures composed of user equipment, UAVs, and high-altitude platforms further model computation offloading as a multi-layer optimization problem that jointly minimizes computation delay and energy consumption [17], while UAV-MEC systems integrate trajectory optimization and resource allocation to reduce mobile user energy consumption and ensure long-term system stability [18]. More recently, computation offloading has been extended beyond purely technical considerations to include economic and strategic factors, such as contract-based incentive mechanisms in wireless-powered and backscattering-assisted MEC systems [19], and game-theoretic resource allocation models in vehicular edge computing environments that incorporate pricing strategies, user preferences, and review information [20]. In addition, energy-aware and low-latency routing models have been proposed to jointly consider data transmission and computation offloading, highlighting energy-centric MEC system design as an emerging research direction [21].

In parallel, deep reinforcement learning (DRL) has been widely adopted as a powerful decision-making framework for complex systems characterized by high-dimensional state spaces, nonlinear dynamics, and uncertain environments. Early investigations demonstrated the applicability of reinforcement learning to hierarchical control problems, such as chemical process control, where DRL was employed to support decision-making across multiple control layers and extended toward multi-agent architectures and standardized development paradigms for industrial deployment. Shifting toward decentralized or distributed computation offloading mechanisms, where edge devices independently determine their offloading decisions based on locally observable information [22], Chen et al. proposed a decentralized DDPG-based framework to jointly minimize power consumption and buffering delay at each user [23], while Pham et al. studied decentralized computation offloading and resource allocation in multi-server MEC systems through joint optimization of transmission power and server-side computation resources [24]. These works primarily focus on continuous offloading control under scheduled or contention-free access assumptions and typically rely on smooth latency or cost models to facilitate learning. While some decentralized approaches incorporate user association or server selection decisions, they generally abstract away MAC-layer access dynamics and assume reliable transmission once an offloading decision is made. As a result, the impact of grant-free access contention, collision-induced retransmissions, and decoding constraints on the learning process remains largely unexplored. This gap motivates the present work, which explicitly integrates grant-free access dynamics into decentralized reinforcement learning for computation offloading. Furthermore, recent studies have widely adopted actor–critic and DDPG-based frameworks for continuous offloading control in MEC systems. However, it has been noted that DDPG is sensitive to reward discontinuities, hyperparameter tuning, and non-stationary environments, which may lead to unstable convergence in systems with abrupt state transitions [25]. Moreover, existing decentralized DDPG-based offloading works typically assume smooth latency models and scheduled access, abstracting away MAC-level contention effects [26], thereby limiting their applicability under grant-free access with collision-induced dynamics.

Beyond algorithm comparison, DRL has been increasingly applied to real-world control systems with multiple objectives and operational time scales. For instance, multi-timescale reward-based DRL frameworks have been proposed for energy management in regenerative braking energy storage systems, demonstrating the importance of reward design in coordinating short-term and long-term control objectives [27]. In large-scale and highly interactive environments, such as connected autonomous vehicle platooning, cooperative multi-agent DRL systems have been developed to exploit vehicle-to-vehicle and vehicle-to-infrastructure communications, enabling agents to collaboratively optimize traffic efficiency, fuel consumption, and system stability [28]. Complementing these application-driven studies, comprehensive survey works have systematically reviewed DRL applications in MEC-enhanced terrestrial wireless networks, encompassing IoT and vehicular networks, and have identified DRL as a key enabler for intelligent resource allocation, task offloading, and network control in next-generation edge computing systems [29].

The multi-agent deep reinforcement learning (MADRL) has also been recently introduced to enable distributed cooperation among multiple devices, where each agent learns its own policy while jointly optimizing a global objective [30,31]. The MADRL framework allows edge devices to make adaptive offloading decisions in dynamic and partially observable environments, where centralized coordination is infeasible. Furthermore, by leveraging centralized training and decentralized execution (CTDE) architectures, agents can effectively learn cooperative policies that minimize task-completion latency while maintaining scalability. In parallel, there is growing interest in grant-free random access for massive machine-type communications, which eliminates explicit scheduling requests and significantly reduces signaling overhead [32,33]. However, integrating grant-free access with decentralized offloading introduces new challenges because multiple devices may attempt simultaneous transmission, leading to collisions and uncertain task delays. Existing works such as the Access Reservation Protocol (ARP) [10] and grant-free multiple access (GFMA) [34] strategies have attempted to manage contention, but these are primarily designed for centralized systems.

Hence, in this paper, we propose a fully decentralized offloading decision strategy based on multi-agent deep Q-network (MADQN) for a multi-user MEC system. Compared with centralized approaches, the proposed decentralized architecture avoids global state aggregation and coordination overhead, thereby avoiding excessive signaling overhead and enabling scalable operation in dense edge deployments. Moreover, unlike existing policy-gradient-based methods that are sensitive to reward discontinuities and strongly coupled agent interactions, the value-based MADQN framework exhibits stable convergence under the structured latency variations induced by grant-free access collisions. These properties make the proposed approach well suited for dense MEC environments with contention-driven dynamics.

Specifically, the main contributions of the paper are summarized as follows:

- We propose a decentralized MADQN-based computation offloading framework that explicitly integrates grant-free access dynamics, including access contention, collision-induced retries, and MAC-level latency effects, into the learning process.
- Through simulation under comparable system settings, the proposed approach compared with benchmark schemes achieves up to 20% reduction in average task latency with ARP and 45% with GFMA, particularly under moderate to high user densities.
- The proposed framework demonstrates faster convergence and more stable learning behavior than baseline offloading strategies, maintaining non-zero offloading decisions across a wide range of contention levels.
- By adopting a fully decentralized architecture, the proposed method avoids centralized coordination overhead and scales efficiently with the number of devices, while remaining robust to the non-stationary and discontinuous latency dynamics induced by grant-free access.

The primary methodological contribution of this work lies in the system–learning co-design that embeds grant-free access dynamics directly into the decentralized reinforcement learning framework. Rather than treating computation offloading and wireless access as separable components, the proposed design jointly models access contention, collision-induced retries, and computation latency within the reward feedback, enabling stable decentralized learning under discontinuous and strongly coupled system dynamics.

Table 1 summarizes key architectural and operational differences to further clarify the distinction between the proposed framework and prior decentralized DRL-based offloading approaches. The comparison highlights differences in access assumptions, MAC-level modeling, reward characteristics, and learning stability considerations.

The rest of this paper is organized as follows. Section 2 presents the overall system model of the considered multi-user MEC network, including the local and remote computation models, channel assumptions, and latency formulations. Section 3 introduces the proposed decentralized offloading framework and the operation procedure under the grant-free access mechanism. Further, we formulate the offloading optimization problem and describe the proposed multi-agent deep Q-network (MADQN) algorithm, detailing its state, action, and reward structures. Section 4 provides the numerical evaluation and performance comparison of the proposed scheme against conventional centralized and grant-free multi-channel access baselines. Finally, Section 5 concludes the paper with key findings and outlines future research directions on scalable decentralized learning for large-scale MEC systems.

## 2. System Model

Table 2 summarizes the key notations and parameters used throughout the system model, latency formulation, and MADQN framework. For clarity and consistency, each symbol is defined once and used uniformly across all sections of the manuscript.

We consider a multiple-input multiple-output (MIMO) system in which a single base station (BS) equipped with *J* antennas is integrated with a multi-access edge computing (MEC) server to provide remote computational capabilities to edge devices (EDs). Let *K* denote the number of edge devices in the system that need the help of the MEC server in executing its task. We assume that each ED is equipped with multiple *I* antennas. Let J and I represent the sets of antenna indices of the BS and each ED, where $J=\left\{1,\cdots ,J\right\}$ and $I=\left\{1,\cdots ,I\right\}$, respectively, and |I|=I and |J|=J.

We consider a scenario where each ED generates a relatively large amount of data. Let $\alpha_{k}$ denote the amount of data that the ED *k* (or, equivalently, the *k*-th ED) generates. Even though each ED has sufficient computation capability, it is willing to engage in task offloading to reduce its task completion latency further. We assume that the data can be arbitrarily divided into multiple segments, and some are executed by on-board local computing while others are executed by remote computing at the MEC server after task offloading, simultaneously.

We denote a channel coefficient matrix between the ED *k* and the BS as $H_{k}=\left[h_{j,i}^{k}\right]\in C^{J\times I}$, and $h_{j,i}^{k}$ denotes the element of the *j*-th row and the *i*-th column, where j∈J and i∈I. Here, $h_{j,i}^{k}$ is the channel coefficient between the *i*-th antenna of the ED *k* and the *j*-th antenna of the BS. Additionally, we consider a block fading channel model where the channel coefficient remains constant during each transmission round, but varies with respect to different ones. For a channel fading coefficient, we assume Rayleigh fading where $h_{j,i}^{k}$ follows a Gaussian distribution with zero mean and unit variance (i.e., $h_{j,i}^{k}∼\mathrm{CN}\left(0,1\right)$). We denote $\gamma_{k}$ as the signal-to-noise ratio (SNR) of the ED *k* with $\gamma_{k}\leq \gamma_{\max}$, where $\gamma_{\max}$ denotes the maximum achievable SNR.

$\beta_{k}$ denotes the offloading factor of the ED *k*, with values ranging from 0 to 1 (i.e., $0\leq \beta_{k}\leq 1$), which implies the portion of data to be offloaded to the MEC server to engage in remote computation. Note that when the offloading factor calculated by the device comes out as zero (i.e., $\beta_{k}=0$), it would engage in local computing only. The ED *k* attempts to initialize offloading by transmitting the first packet of data to the BS without any grant acquisition procedure (i.e., a grant-free manner). Since the BS is equipped with *J* antennas, it can decode at most *J* individual streams at the same time. Let *n* denotes the number of EDs attempting offloading at the same time (i.e., n≤k). Then, the first packet from the ED *k* can be successfully decoded when the number of devices attempting to transmit the packet is less than or equal to *J* (i.e., n≤J).

For the ED *k* with data $\alpha_{k}$ and offloading factor $\beta_{k}$, the amount of data to be offloaded is calculated as $\alpha_{k}\times \beta_{k}$, whereas its local data is computed as $\alpha_{k}\times \left(1-\beta_{k}\right)$. Accordingly, its local computation latency denoted by $T_{k}^{l}$ can then be calculated as

(1) $$T_{k}^{l}=\frac{d_{k}\times \alpha_{k}\times \left(1-\beta_{k}\right)}{f_{k}},$$

where $d_{k}$ is the number of CPU cycles required to process 1-bit of data at the ED *k* and $f_{k}$ is the computing capability of the ED *k*. Note that $T_{k}^{l}$ is a function of $\beta_{k}$.

The latency for the remote computation can be basically calculated as the summation of the latency during the offloading, the latency required for remote computation, and the latency for receiving the remote computation result from the server. Note that we consider a grant-free initial attempt for the offloading, and several reattempts can occur due to failure. Thus, the latency spent during the reattempts may be considered. Let $T_{k}^{r}$ denote the latency for the remote computation, and it can be represented as

(2) $$T_{k}^{r}=T_{k}^{\mathrm{initialization}}+T_{k}^{\mathrm{offloading}}+T_{k}^{\mathrm{computing}}+\epsilon ,$$

where $T_{k}^{\mathrm{initialization}}$, $T_{k}^{\mathrm{offloading}}$, $T_{k}^{\mathrm{computing}}$, and ϵ indicate the latency incurred during the reattempts, the latency during the offloading, the latency required for remote computation, residual delay term accounting for result-return or control overhead after remote computation, respectively.

In (2), we define the latency incurred during the reattempts as follows:

(3) $$T_{k}^{\mathrm{initialization}}≜T_{S}+\left(m-1\right)\times T_{W},$$

where *m* is the number of initialization attempts, $T_{S}$ and $T_{W}$ indicate the time duration for the success of the reattempt and the waiting time before a reattempt is made, respectively. Note that $T_{W}$ becomes effective when reattempt fails more than once (i.e., m>1). The latency incurred by access contention and collision resolution at the MAC layer is reflected in the re-attempts and waiting time components of the initialization latency. The detailed grant-free access and reattempt procedure is described in Section 3.1.

In (2), we define the latency during the offloading as follows:

(4) $$T_{k}^{\mathrm{offloading}}≜T_{0}\times n_{k}^{\mathrm{offloading}},$$

where $T_{0}$ denotes the time duration for a time slot during a transmission round and $n_{k}^{\mathrm{offloading}}$ indicates the number of transmissions required to transmit the data amount of $\alpha_{k}\times \beta_{k}$ offloaded to the BS. Specifically, $n_{k}^{\mathrm{offloading}}$ is the minimum number that satisfies $\sum_{t=1}^{n_{k}^{\mathrm{offloading}}}R_{k}\left[t\right]\;\geq \;\alpha_{k}\times \beta_{k}$ for the achievable data rate at time slot *t*, where it is given by

(5) $$R_{k}\left[t\right]=B_{k}\times \sum_{l=1}^{m_{min}}\log_{2}\left(1+\gamma_{k}\times \lambda_{l}^{2}\right),$$

where $B_{k}$ denotes the bandwidth allocated to the ED *k*, $m_{min}=\min\left(I,J\right)$, and $\lambda_{l}$ is the *l*-th eigenvalue after scalar value decomposition (SVD) of $H_{k}$. Note that we consider $B_{k}=B\;\forall k\in K$ since each device uses the total bandwidth *B* during offloading. For analytical clarity, we assume equal bandwidth allocation among offloading devices. This abstraction allows us to isolate the impact of access contention and offloading decisions on latency. In practice, unequal bandwidth allocation would primarily scale transmission delays and introduce additional heterogeneity, but would not change the qualitative congestion dynamics or the structure of the learning problem.

In (2), the latency required for remote computation at the MEC server can be derived as

(6) $$T_{k}^{\mathrm{computing}}=\frac{d_{s}\times \alpha_{k}\times \beta_{k}}{f_{s}},$$

where $f_{s}$ and $d_{s}$ denote the maximum computation capability (cycles/sec) of the MEC server and the required number of CPU cycles needed for processing 1-bit of data at the MEC server, respectively.

Finally, in (1) and (2), the total amount of time the ED *k* spends in successfully executing an amount of data generated is given as

(7) $$L_{k}=\max\left(T_{k}^{l},\;T_{k}^{r}\right).$$

Equation (7) adopts a max-based latency model, which assumes that local computation and the remote execution pipeline can proceed concurrently after the offloading decision is made. Although some offloading stages are inherently sequential in practice, this abstraction is widely used to capture the dominant latency bottleneck. While a more detailed sequential model would increase absolute latency values, it would not alter the qualitative behavior of the proposed MADQN-based offloading policy.

Furthermore, to characterize the reliability of grant-free offloading under contention, we define the offloading success probability as

(8) $$P_{\mathrm{succ}}=\frac{N_{\mathrm{succ}}}{N_{\mathrm{off}}},$$

where $N_{\mathrm{off}}$ denotes the total number of offloading attempts and $N_{\mathrm{succ}}$ is the number of attempts successfully decoded within the decoding limit *J* and maximum retransmission constraint *m*.

The proposed framework is particularly suitable for dense uplink MEC scenarios such as multi-user augumented reality (AR)/virtual reality (VR) and industrial IoT systems, where a large number of devices generate sporadic, latency-sensitive computation tasks. The considered grant-free access model is aligned with 5G NR configured grant–based uplink transmission, in which devices transmit without per-packet scheduling requests. The base station follows standard contention detection and decoding procedures, while the proposed MADQN-based decision-making is executed entirely at the device side, requiring no modification to existing 5G NR base-station protocols.

Furthermore, from an implementation standpoint, the proposed framework incurs minimal operational overhead. Since each device makes offloading decisions using only local observations, no additional signaling or coordination messages are introduced beyond standard grant-free uplink transmissions. The learning model can be trained offline and deployed for online inference, with device-side computation limited to lightweight DQN forward evaluation, making the approach practical for resource-constrained edge devices.

In this section, we have presented the system model of the considered multi-user MEC network, including the local and remote computation processes, wireless channel assumptions, latency components, and specific scenario where the proposed model offers a measurable benefit. The system model highlights that the task completion latency of each edge device depends on various dynamic factors, such as channel quality, computing capacity, and the number of simultaneous offloading attempts. Because these parameters vary over time, determining the optimal offloading decision through analytical methods is challenging. Therefore, in the following section, we introduce a framework to develop a decentralized offloading decision strategy that allows each edge device to learn its optimal offloading behavior autonomously.

## 3. Decentralized Offloading Strategy

In this section, we present the proposed decentralized offloading framework based on multi-agent deep reinforcement learning. For clarity, the MADQN architecture adopted in this work follows a standard value-based multi-agent formulation. The novelty of the proposed approach as stated in the introduction lies in the joint modeling of grant-free access dynamics and decentralized offloading decisions, rather than in proposing a new learning algorithm. The main objective is to enable each edge device to autonomously determine its optimal offloading behavior in a dynamic and partially observable MEC environment. Unlike centralized schemes that require global coordination, the proposed approach operates in a fully decentralized manner, where devices make decisions using only their locally available information, such as channel state and the number of active users. To achieve this, we design a learning-driven framework that models the offloading problem as a sequential decision process and employs a multi-agent deep Q-network (MADQN) to learn adaptive offloading policies through interaction with the environment. The following subsections describe the decentralized operation procedure, formulate the optimization problem, and detail the MADQN architecture and training methodology used to realize the proposed strategy.

### 3.1. Overview of the Decentralized Offloading Process

**Figure 1:**
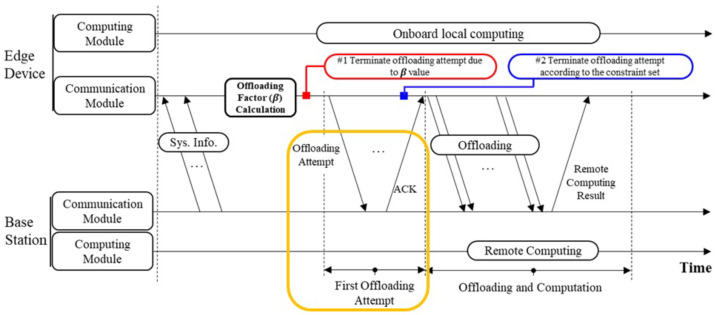
The overall operation procedure of the decentralized offloading process.

The overall operation procedure of the decentralized offloading process is described in Figure 1. Each active ED with its generated data deciding to engage in offloading calculates its $\beta_{k}$ based on its local observation. If the offloading factor calculated by the ED *k* is greater than 0, the ED *k* then prepares to engage in offloading. Otherwise, the ED *k* automatically engages in local computation without having the option to try again. Therefore, all active devices with their β values greater than 0 prepare to engage in offloading whereas all others engage in local computation. Unlike traditional BS-controlled scheduling, this distributed process allows simultaneous access attempts without explicit coordination, significantly reducing control signaling overhead. However, it also introduces potential collisions among users that must be learned and mitigated through adaptive decision policies.

The active EDs with $\beta_{k}>0$ are termed offloading devices, and the total number of offloading devices in the system at a particular time slot is denoted by the parameter *n*. These *n* devices simultaneously attempt offloading to the MEC server. If *n* is less than or equal to the number of antennas at the BS (i.e., n≤J), it implies the BS can service all the *n* EDs at the time. Therefore, all *n* receives an acknowledgment (ACK) from the BS and continuously proceed with either their partial or full offloading and finally remote computation. This condition reflects the spatial multiplexing limit of the multi-antenna BS, indicating that the system’s immediate throughput depends on both *n* and *J*, which dynamically vary with user activity.

However, if the condition is not satisfied (i.e., n>J), all the offloading devices (*n*) attempting to offload their task to the BS fails at the same time since the BS has a limited decoding capability, depending on the number of antennas. This implies that the number of devices attempting to offload at the same time exceeds the number of devices the BS could handle. It indicates the number of packets received being an important factor for the decoding. In this case, the BS sends a broadcast of negative acknowledgment (NACK), which is received by all devices. Upon receiving the NACK, the offloading devices stop all activities and reattempt the whole process again (i.e., calculating their offloading factor and offloading at the next available time). Such repetitive contention and reattempt behaviors form the stochastic environment that our learning algorithm must adapt to, making the decentralized learning-based approach particularly effective in dynamically balancing local and remote workloads.

The number of reattempts of an ED *k* is denoted by the parameter *m*, and all failed offloading devices have the opportunity to reattempt the entire process several times until the maximum number of attempts ($M_{max}$) required is reached. If a device still fails to offload to the BS even after $M_{max}$ is achieved, the device terminates all actions leading to remote computation and focuses on local computation only.

Although all devices experience a failed access attempt when the number of simultaneous offloading users exceeds the BS decoding capability, this does not lead to degenerate learning behavior. Successful offloading events occur when the number of contenders is below the decoding limit, yielding significantly lower latency than local execution. Moreover, the use of partial offloading allows agents to adopt conservative offloading strategies under high contention. As a result, the learned policy balances the trade-off between offloading gain and access risk, rather than converging to a trivial never-offload solution. This behavior is further reflected in the non-zero offloading factors and success probability observed across a broad range of user densities in the simulation results.

### 3.2. Interaction Between Grant-Free Access and Learning Dynamics

While the above section details the decentralized offloading procedure, we clarify how grant-free access outcomes influence the learning process in this section. Under grant-free transmission, simultaneous access attempts may exceed the decoding limit, leading to collisions, retransmissions, or enforced waiting. These outcomes do not alter the action space or state transition rules explicitly, but manifest as increased execution latency, which is reflected in the reward signal. As a result, repeated access failures induce negative reward feedback, biasing agents toward more conservative offloading decisions under congestion and more aggressive offloading when contention is low. The retransmission limit *m*, waiting time $T_{W}$, and decoding capacity *J* jointly determine the severity and persistence of contention-induced penalties. Larger values of *m* or $T_{W}$ increase the latency cost of repeated access failures, while a smaller decoding limit *J* raises the collision probability under high user density. Through the reward signal, these parameters shape the agent’s long-term policy adaptation.

### 3.3. Problem Formulation

Due to shared wireless and computational resources, the task completion latency experienced by each edge device is inherently coupled with the offloading decisions of other devices. In particular, the latency $L_{k}$ of device *k* depends not only on its own offloading factor $\beta_{k}$, but also on the number of simultaneously offloading devices, which emerges from the collective behavior of all agents.

We define a decentralized learning objective in which each edge device aims to minimize its own expected task completion latency through repeated interaction with the environment. The system dynamics, including access contention and the decoding capability of the BS, are governed by physical constraints and are reflected in the environment’s state transitions and reward feedback. Hence, constraints such as the maximum number of decodable streams at the BS and the maximum number of offloading attempts are treated as environmental dynamics rather than explicit decision constraints.

Under this formulation, each device learns an offloading policy that maps its local observations to an offloading action, with the goal of minimizing long-term latency in a coupled multi-agent environment. Accordingly, the following expressions define the performance objective and system dynamics considered in our learning framework:

(9) $$\left(P1\right)\min_{\beta_{k}}\;L$$

(10) $$\begin{array}{cc} \mathrm{such}\mathrm{that} & n(t)\leq K(t),\forall t\in T, \end{array}$$

(11) $$\begin{array}{cc} \; & n(t)\leq J,\forall t\in T, \end{array}$$

(12) $$\begin{array}{cc} \; & m\left(t\right)\leq M_{max},\forall t\in T, \end{array}$$

where constraints (10) and (11) ensures that the number of devices engaging in offloading at a particular time slot does not exceed the total number of devices in the system as well as the number of receive antennas at the BS, and the last constraints (12) ensures that the number of attempts of a particular device does not exceed the required maximum number of attempts. Note that (10)–(12) characterize system dynamics and physical limitations of the MEC environment rather than controllable constraints imposed on individual agents. They jointly maintain system feasibility, preventing channel overload and ensuring fairness among devices that share limited spatial and temporal resources.

Our proposed formulated problem (9) is a function of $\beta_{k}$. Hence, we aim to find the optimal offloading factor of each device that reduces or minimizes its task completion latency. However, since both $\beta_{k}$ and the number of active users *n* vary over time in a stochastic and interdependent manner, finding an analytical solution is intractable. Therefore, the offloading problem is reformulated as a sequential decision-making process where each device acts as an autonomous agent interacting with a time-varying environment. This transformation enables the use of reinforcement learning to derive adaptive policies that minimize long-term task latency under uncertainty.

Each ED calculates its optimal offloading factor based on its local observation. We assume that the BS sends a broadcast of the number of active devices (*K*) in the system to all the devices at the beginning of each time slot. This information can be conveyed using a low-rate control signal similar to existing load or access barring indicators in cellular systems. Since only a scalar value is broadcast once per slot, the signaling overhead is negligible compared to data transmission and does not affect system latency. These devices then obtain their channel state information ($H_{k}$) and *K*, broadcasted by the BS. Based on these two pieces of information, the EDs calculate the amount of data that should be offloaded to the MEC and the amount that should be computed locally that generates the minimum task completion latency.

### 3.4. Multi-Agent Deep Q-Network (MADQN) Design and Training

**Figure 2:**
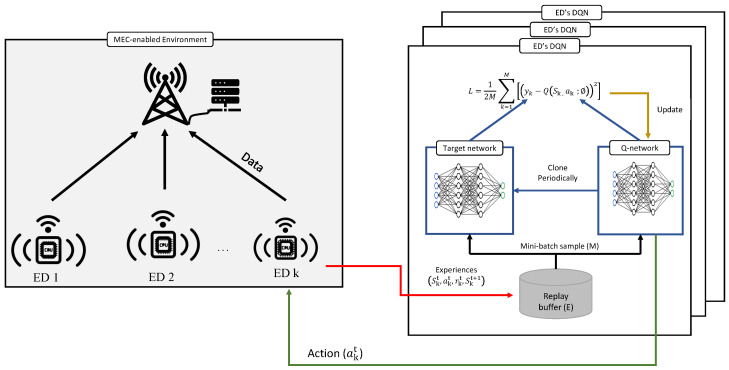
An illustration of the decentralized environment and DQN agents.

For our objective in (9), we adopt a multi-agent deep Q-neural network (MADQN) algorithm, an off-policy reinforcement learning method that trains an agent to produce an action based on the device’s local observation. This choice is motivated by the highly non-stationary, discontinuous environment induced by grant-free access, in which collision events lead to abrupt, collective latency penalties. In such settings, value-based methods have been shown to exhibit more stable convergence than policy-gradient approaches, which are sensitive to reward discontinuities and coupled agent behavior. Importantly, the performance gains observed in this work are primarily attributable to the integration of grant-free access dynamics into the system and reward modeling, while the choice of a value-based learner mainly contributes to robustness under discontinuous feedback rather than algorithmic superiority. Figure 2 shows an illustration of the decentralized environment and DQN agents. In this design, each edge device is modeled as an independent learning agent that interacts with the environment to refine its policy over time. By observing its own channel condition and system load, each agent learns to determine an appropriate offloading factor that optimizes its individual latency while collectively improving network efficiency. We briefly describe the main components (i.e., observation, agent, action, reward) of the proposed MADQN algorithm as follows:

- **Observation or State Space**: The state space defines the representation of our environment from each device *k*’s perspective, which will be used to determine the value of $\beta_{k}$. For decentralized policies, each device’s state space only depends on its local observations. The MADQN agent makes use of either a continuous or discrete observation space, which in our case is |H| and *K*. In summary, the state space from the perspective of an ED *k* at slot *t* is defined as
  (13) $$s_{k}^{t}=\left[\right|H_{k}\left(t\right)\left|,K\left(t\right)\right],$$
  where $|H_{k}\left(t\right)|$ represents the instantaneous channel strength affecting transmission success and K(t) indicates the number of concurrently active users.
- **Agent**: DQN agents are value-based reinforcement learning agents that train Q-networks to estimate the expected value of discounted cumulative long-term rewards, and therefore do not make use of actor networks. DQN is an improvement or modified form of the Q-learning algorithm. We consider that each device *k* has an agent to exploit a decentralized policy. Thus, we assume that the number of devices in the system (i.e., *K*) equals the number of DQN agents. These agents are responsible for determining the value of $\beta_{k}$ of the devices based on their observations. As training progresses, each agent independently updates its Q-network using its own experiences, which allows scalable and fully decentralized learning without parameter sharing or explicit coordination.
- **Action Space**: The DQN agent makes use of a discrete action space. Based on the current state of the system observed by each device’s agent, an agent selects an action to adjust the offloading factor that determines the user association and the amount of data to be offloaded to the MEC server and computed locally at the ED. For each agent, the action will be selected for each time slot t∈T, and it is denoted as follows:
  (14) $$a_{k}^{t}=\beta_{k}\left(t\right).$$
  Note that discrete quantization of $\beta_{k}$ enables robust learning while preserving sufficient flexibility to capture meaningful offloading decisions. That is, it enables faster convergence and simplifies implementation, while still capturing the essential trade-off between local and remote computing.
- **Reward Function**: Each edge device operates as an autonomous learning agent whose behavior is guided by a scalar reward signal. The design of this reward is therefore critical, as it directly influences both the convergence behavior and the quality of the learned offloading policy. To enable agents to adapt their decisions over time and reduce the cumulative computation cost defined (7), we define the instantaneous reward obtained by device *k* at time step *t* as follows:
  (15) $$r_{k}^{t}=-L_{k}.$$
  This negative reward formulation ensures that minimizing latency directly corresponds to maximizing cumulative reward, thereby aligning individual agent objectives with the system-wide performance goal.

The main idea in many RL algorithms is to estimate the value of the optimal policy or action-value function by iteratively updating its values at each time step. DQN agents use two parameterized action-value functions, each maintained by a corresponding critic. Having an observation space s and an action a, the Q-network function $Q\left(s_{k},a_{k};\phi \right)$, following the optimal policy, stores the corresponding expected discounted cumulative long-term reward estimate. To further improve the stability of the optimization, the agent periodically updates the target network parameters $Q_{t}\left(s_{k},a_{k};\phi_{t}\right)$ using the latest Q-network parameters. Where ϕ and $\phi_{t}$ are the Q-network and target network parameter values. Both the Q-network and target networks have the same structure and parameterization. During training, the parameter values in ϕ are tuned by the agent. These parameters remain at their tuned values after training. The trained value function is approximated and stored in the Q-network $Q\left(s_{k},a_{k}\right)$. In our implementation, experience replay and target network updates are periodically applied to stabilize convergence and avoid divergence during multi-agent learning.

We consider a multi-agent DRL to perform decentralized training for all agents and thus do not incorporate parameter sharing among agents. In other words, each agent uses their own experiences.To train and assess the proposed decentralized offloading framework, we implement a simulation environment in MATLAB Simulink, as illustrated in Figure 2. The environment emulates the interaction between edge devices and the MEC system, enabling each agent to repeatedly select offloading actions and observe the resulting system response. During both training and evaluation phases, the environment processes the agents’ decisions and provides corresponding reward feedback, which is used to update the learned policies. During testing, the trained Q-networks are fixed, and each agent selects the greedy action corresponding to its observed state, allowing fully decentralized execution without the need for inter-agent communication.

Algorithm 1 illustrates the details of the training procedure. To have a better exploration performance, our interactions are randomly started with a random initial state $s_{k}^{1}$ for each *k*. The interactions are terminated after a predefined maximum step $T_{max}$ for each episode. At each time slot *t* during an episode, each agent’s experience tuple $\left(s_{k}^{t},a_{k}^{t},r_{k}^{t},s_{k}^{t+1}\right)$ is stored in an experience replay buffer *E*. Then, the agent’s critic network is updated by randomly sampling a mini-batch of experience tuples ${\left(s_{m},a_{m},r_{m},s_{m}^{\prime }\right)}_{m=1}^{M}$ from *E*. The parameters of the Q-network are thus regularly copied to the target network. At each time step, the Q-network parameters are updated to minimize the loss function as follows:

(16) $$L=\frac{1}{2M}\sum_{k=1}^{M}{\left(y_{k}-Q\left(s_{k},a_{k};\phi \right)\right)}^{2},$$

where $y_{k}=r_{k}+\zeta \times max_{a_{k}^{t+1}}Q_{t}\left(s_{k}^{t+1},a_{k}^{t+1};\phi_{t}\right)$ and ζ is a discount factor. Finally, after training $K_{max}$ episodes, the dynamic computation offloading policy will be gradually and independently learned by each user agent.
**Algorithm 1** Training procedure of the proposed multi-agent deep Q-network (MADQN)-based decentralized offloading framework**  Initialization:**1:**for** each user agent k∈K **do**2:     Initialize learning rate, gradient decay factor, exploration rate3:     Initialize critic $Q\left(s_{k},a_{k};\phi \right)$ and target critic $Q_{t}\left(s_{k},a_{k};\phi_{t}\right)$ network (i.e., $\phi \leftarrow \phi_{t}$)4:     Initialize experience replay buffer *E* with length *C*5:**end for****Training:**6:**for** each $episode=1,2,...,K_{max}$ **do**7:      Randomly generate an initial state $s_{k}^{1}\left(\right|H_{k}\left|,k\right)$ for ∀k∈K8:      **for** each time slot t=1,2,...,T **do**9:          **for** k∈K **do**10:              Determine local and remote execution latency by selecting an action $a_{k}^{t}$,              with probability ε, otherwise select $a_{k}^{t}=argmax_{a}Q\left(s_{k},a_{k};\phi \right)$11:              Execute action $a_{k}^{t}$ and obtain reward $r_{k}^{t}$ and next state $s_{k}^{t+1}$12:              Store in *E* and sample a random mini-batch of *M* experiences $\left(s_{k}^{t},a_{k}^{t},r_{k}^{t},s_{k}^{t+1}\right)$13:              **if** $s_{k}^{t+1}$ is a terminal state **then**14:                  $y_{k}=r_{k}$15:              **else**16:                  $y_{k}=r_{k}+\zeta \times max_{a_{k}^{t+1}}Q_{t}\left(s_{k}^{t+1},a_{k}^{t+1};\phi_{t}\right)$17:              **end if** // For all experiences in *M*18:              Update critic parameters using one-step minimization of the loss in (16)19:              Update target critic $\phi_{t}=\tau \phi +\left(1-\tau \right)\phi_{t}$              // τ indicates the smoothing factor20:          **end for**21:    **end for**22:**end for**

While the proposed framework is evaluated under a grant-free access model with a decoding limit, the decentralized learning structure is not inherently tied to this specific access mechanism. The access model primarily affects the latency and reward computation through contention, collision, and waiting-time terms. Alternative access schemes, such as hybrid scheduled–grant-free or partially coordinated access, can be accommodated by modifying the access-related latency components and reward feedback, while preserving the same state, action, and MADQN learning architecture. In this sense, the learning framework and decentralized decision structure are generally applicable, whereas the access modeling and reward formulation are access-model dependent.

## 4. Numerical Results

In this section, we provide several numerical results to illustrate the performance of our proposed MADQN algorithm for decentralized offloading in our MEC system. Simulation parameters are summarized in Table 3.

For clarity, while the simulations consider identical device parameters, the proposed framework naturally extends to heterogeneous settings with device-specific CPU frequencies, task sizes, and energy coefficients. Such heterogeneity would primarily increase the diversity of local state observations and reward values, but would not alter the underlying access contention mechanism or the decentralized learning dynamics. Since decisions are made locally based on observed latency outcomes, the framework remains applicable under heterogeneous system configurations.

**Figure 3:**
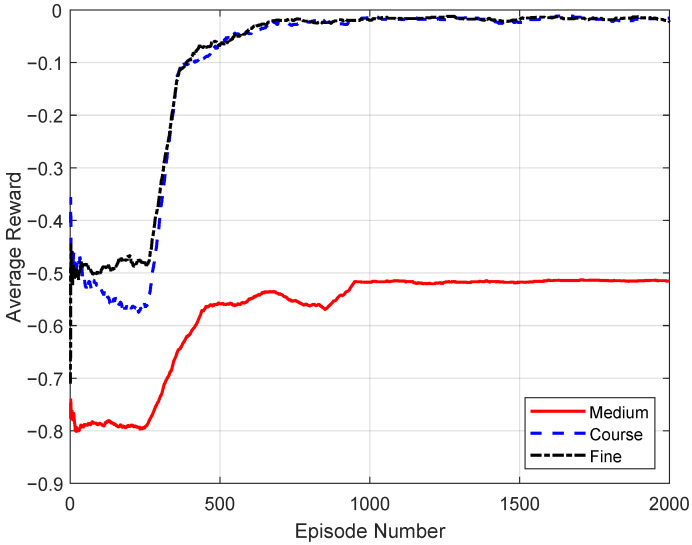
Effect of action-space discretization granularity on MADQN convergence.

Figure 3 illustrates the impact of discretization granularity on MADQN training behavior, where coarse, medium, and fine correspond to offloading-factor discretizations with step sizes of δβ=0.5, 0.1, and 0.05, respectively. Coarser action discretization enables faster convergence due to a smaller action space, but limits the achievable steady-state reward. Finer discretization improves asymptotic performance by allowing more precise offloading decisions, at the cost of increased training complexity and slower convergence. The medium discretization provides a balanced trade-off between convergence speed and control resolution, achieving stable learning with near-optimal steady-state performance. These results justify the selected discretization as a practical balance between learning stability and performance.

Based on this sensitivity analysis, a discretization step size of Δβ=0.1 is adopted in the remainder of the paper, as it provides a favorable balance between convergence speed, learning stability, and steady-state performance.

**Figure 4:**
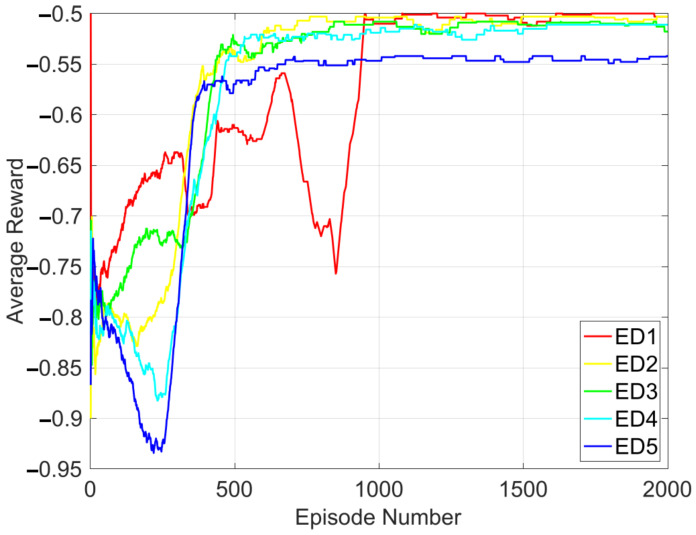
Average reward during training with J=8 and K=5.

Figure 4 shows a graph of the average reward per episode against a total of 2000 interactions between the user agents and the environment (i.e., the number of episodes, $K_{max}=2000$), considering J=8 and K=5. For each edge device representing each agent, the average reward increases as the interaction between the agents and the proposed environment increases. It demonstrates that efficient computation offloading policy is gradually and independently learned by each user agent as required. Furthermore, each learned policy stabilizes after about 1000 episodes.

**Figure 5:**
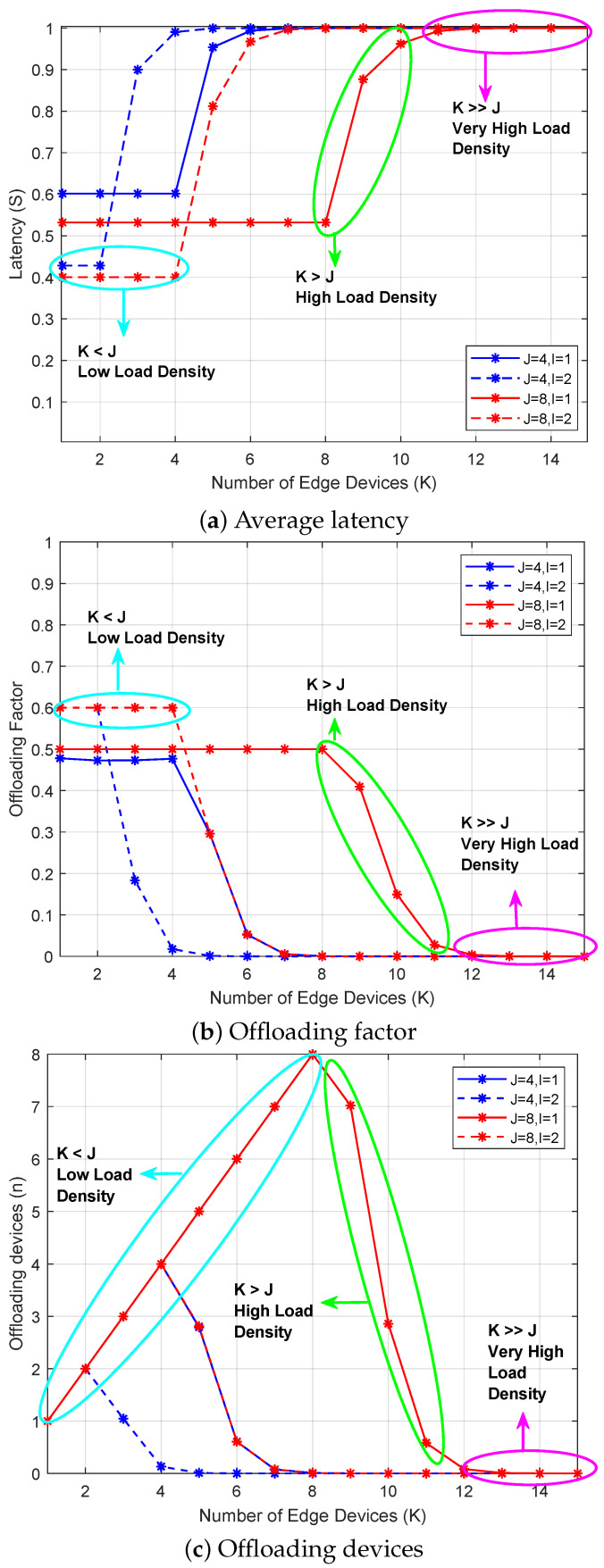
Latency, offloading factor, and the number of offloading devices when varying the number of edge devices.

Figure 5 includes three subfigures that show latency, offloading factor, and the number of offloading devices when varying the number of edge devices. Figure 5a shows the average task-completion latency of the devices for different numbers of antennas (*J*) and transmit antennas (*I*). As *K* increases, the latency also increases and finally converges to 1 s, which corresponds to the maximum latency value achievable when $\alpha_{k}=0.1$ Gbits and the device performs local computation only. This behavior implies that a higher number of users leads to a greater probability of contention and failed offloading attempts, causing more devices to switch to local computation. The minimum latency is achieved when K≤J for all cases, as most devices can successfully complete offloading on their first attempt or within a few reattempts. Furthermore, increasing *J* from 4 to 8 for both I=1 and I=2 enables the BS to serve more devices simultaneously, thereby reducing latency. Similarly, higher transmit antennas (*I*) at the devices provide improved link robustness, which also contributes to lower latency and a higher proportion of successful remote computations.

Figure 5b presents the corresponding average offloading factors of the edge devices for all the different configurations in Figure 5a. The maximum offloading factor is achieved when K≤J, meaning that most devices can successfully access the BS and offload a larger portion of their tasks. As *K* further increases beyond *J*, the offloading factor gradually decreases because only a smaller subset of devices can be accommodated by the BS’s spatial multiplexing limit. This decline indicates that the system becomes more congested, forcing many devices to rely primarily on local computation. In other words, the offloading factor curve directly reflects the access success rate under varying user densities and antenna configurations, validating the MADQN agents’ ability to adaptively reduce their offloading load when the network is crowded.

Figure 5c shows the number of active offloading devices corresponding to each configuration in Figure 5a. It can be observed that *n* is always less than or equal to *J*, confirming that the BS’s decoding capacity is never exceeded. As *K* increases, *n* initially grows linearly but eventually saturates at *J*, after which additional devices are unable to access the BS simultaneously. This saturation behavior demonstrates the effect of the grant-free access constraint and shows that the learned decentralized policy successfully regulates the number of concurrent offloading attempts to maintain system stability. Collectively, the consistent trends in Figure 5a–c confirm that the proposed framework dynamically balances local and remote computation through adaptive offloading decisions learned via MADQN.

**Figure 6:**
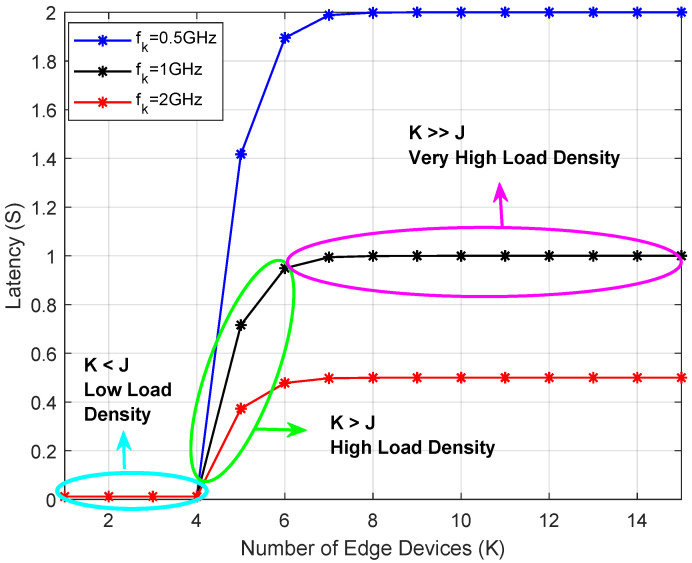
Average latency versus *K* for different device-side computing capabilities $f_{k}$.

Furthermore, to evaluate robustness under heterogeneous device capabilities, we additionally consider multiple device-side computing scenarios with $f_{k}\in \left\{0.5,1,2\right\}$ GHz, representing low-power IoT devices, standard mobile users, and high-performance edge devices, respectively, while keeping the MEC server capability fixed in Figure 6. It could be observed that, increasing $f_{k}$ directly reduces local computation latency, which in turn lowers the overall task completion latency under identical access conditions, demonstrating the robustness of the proposed framework to heterogeneous device capabilities.

**Figure 7:**
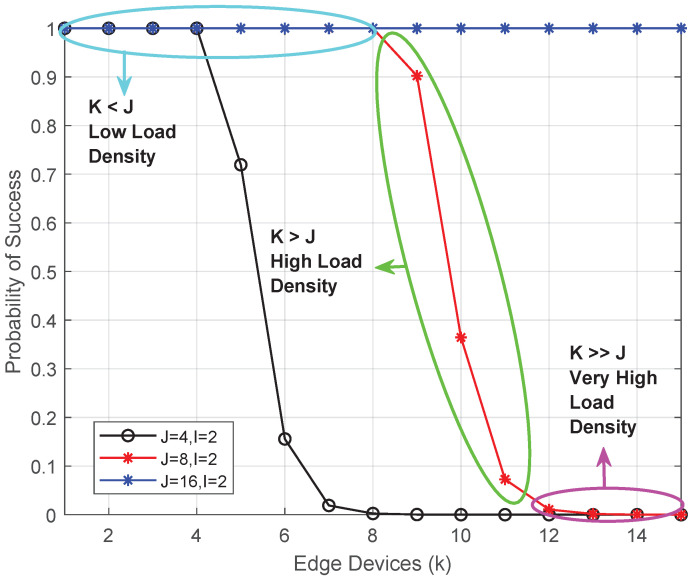
Offloading success probability versus *K* under different decoding limits *J*.

Figure 7 illustrates the offloading success probability under different decoding capacities. When the number K≤J, grant-free transmissions are decoded successfully with high probability. As *K* exceeds *J*, access collisions become dominant, leading to a degradation in success probability. Increasing *J* shifts this transition point to higher user densities, confirming that decoding capacity directly governs contention resilience in grant-free MEC systems. It also indicates that the proposed framework accurately captures the impact of grant-free access constraints on offloading feasibility, and by explicitly modeling decoding limits and contention-induced failures, the learning agent receives structured feedback that enables stable policy adaptation under dense deployment conditions.

The annotated regions in the figures indicate the transition from low-load to collision-dominated regimes as the number of offloading devices exceeds the decoding limit *J*.

**Figure 8:**
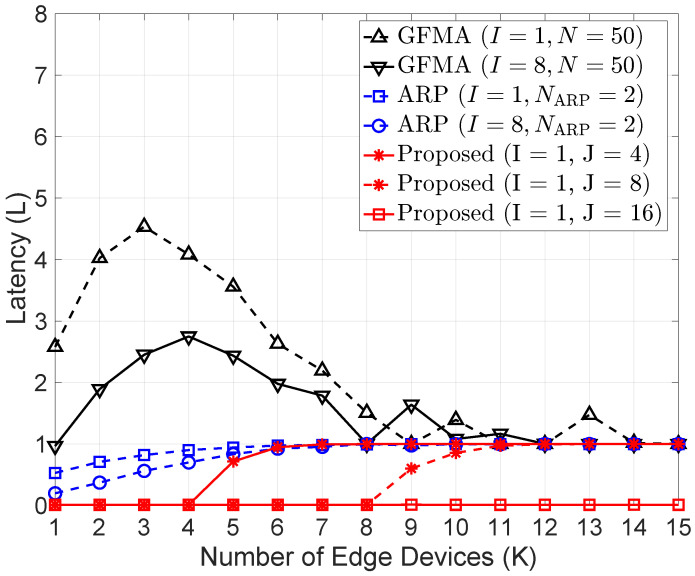
Performance comparison of different offloading decision schemes when varying the number of edge devices.

Figure 8 shows performance comparison of different offloading decision schemes when varying the number of edge devices. For performance comparison in terms of latency, we consider two benchmark schemes, ARP-based offloading decision [10] and GFMA-offloading strategy [34], described as follows:

- **ARP-based offloading (ARP in Figure 8)**: The ARP-based offloading decision is a centralized algorithm that works in a similar manner to our proposed algorithm. However, it is a connection establishment algorithm, named by access reservation protocol (ARP). Basically, the ARP scheme is a contention-based two-step protocol, where devices establish a connection with the BS with the help of an $N_{ARP}$ resource during an access reservation phase and later perform consecutive scheduling-based offloading.
- **GFMA-offloading strategy (GFMA in Figure 8)**: The GFMA-offloading strategy also considers a multi-channel slotted ALOHA protocol for connection establishment between the EDs and the BS. In this scheme, each device randomly selects a resource from an *N* number of resources whenever it attempts to send a portion of its partitioned task.

In Figure 8, we set the simulation parameters of all three algorithms to be equal for a fair comparison. When K=3, the ARP-based algorithm had an average latency ranging from 0.5 to 1 s for different values of transmit antennas (*I*). However, our decentralized algorithm had a very low latency of about 0.01 s, whereas the GFMA-based strategy had a high latency of 2.5 and 4.5 s. Moreover, the ARP-based algorithm converges quickly at 1 s, the maximum attainable latency, when K=6 for I=8 and $N_{ARP}=2$, whereas our proposed decentralized algorithm converges when K=12 for J=8. At moderate user densities, the proposed scheme consistently outperforms ARP and GFMA, reducing average task latency by approximately 20% and 45% respectively, which indicates that our proposed decentralized algorithm is efficient and outperforms the already existing centralized algorithm.

### Limitations and Discussion

The proposed framework relies on accurate local observations, such as channel and task-related parameters. While estimation errors or delayed measurements may introduce uncertainty, learning is driven by observed execution latency, making the approach relatively robust to moderate inaccuracies, albeit with potentially slower convergence. The framework further assumes slowly varying traffic and channel conditions; abrupt exogenous non-stationarities may temporarily degrade performance and motivate future extensions based on continual learning or adaptive exploration. Additionally, agents learn independently without explicit coordination, which enhances scalability but may lead to suboptimal outcomes in extremely dense scenarios where limited inter-agent coordination could further reduce contention. Finally, although discretizing the offloading factor improves learning stability, it may limit optimality when fine-grained control is required. In particular, if the optimal decision lies between discretization levels or when system conditions vary rapidly, discretized action selection may lead to conservative behavior. Increasing the discretization resolution trades off computational complexity and convergence speed, and the chosen discretization provides a practical balance between performance and robustness in the considered scenarios.

## 5. Conclusions

In this paper, we propose a grant-free multi-agent deep Q-network (MADQN)-based decentralized offloading strategy for multi-user multi-access edge computing (MEC) systems. The proposed framework enables each edge device to autonomously determine its offloading factor using only local observations, thereby minimizing task completion latency without requiring centralized coordination. Simulation results verified that the proposed approach achieves significantly lower latency and faster convergence compared to conventional centralized and grant-free baseline schemes. The proposed strategy demonstrates the effectiveness of decentralized learning in dynamic and large-scale MEC environments.

Future work will extend the proposed framework to energy-aware and multi-server MEC scenarios by augmenting the reward function to jointly capture latency and energy consumption through weighted or constraint-based formulations and by extending the state and action spaces to include server-load indicators and server-selection decisions. In addition, while the current framework adopts fully independent learning for scalability, joint or federated multi-agent reinforcement learning could be incorporated through periodic parameter sharing or model aggregation to mitigate non-stationarity, improve stability, and reduce training overhead in dense deployments.

## Figures and Tables

**Table 1:** Comparison between the proposed approach and representative decentralized DRL-based computation offloading methods.

Aspect	Typical DDPG-Based Approaches	Discrete DQN-Based Approaches	Proposed MADQN Framework
Action space	Continuous	Discrete	Discretized partial offloading factor
Access mechanism	Scheduled, contention-free	Scheduled, contention-free	Grant-free access with contention
MAC-level modeling	Not explicitly modeled	Abstracted or simplified	Explicitly incorporated via retries and waiting delays
Collision handling	Often ignored or abstracted	Often ignored	All-or-nothing collision with reattempts
Reward continuity	Smooth, continuous	Piecewise	Discontinuous due to access failures
Learning stability under contention	Not addressed	Limited	Explicitly analyzed and discussed
System dynamics	Weakly coupled agents	Weakly coupled agents	Strongly coupled via shared access channel
Design focus	Algorithm-centric	Algorithm-centric	System–learning co-design

**Table 2:** Parameters and notation.

Parameter	Meaning
*k*	Index of edge device (ED)
*t*	Time index (slot)
*K*	Number of edge devices
*I*	Number of antennas at the ED
*J*	Number of antennas at the BS
I	Set of antenna indices of the ED
J	Set of antenna indices of the BS
$\alpha_{k}$	Data size generated by ED *k*
$\beta_{k}$	Offloading factor of ED *k*
Δβ	Discretization step size
*n*	Number of offloading devices in a time slot
*m*	Number of initialization attempts
$M_{\max}$	Maximum number of initialization attempts
$H_{k}$	Channel matrix between ED *k* and the BS
$h_{j,i}^{k}$	Channel coefficient from antenna *i* of ED *k* to antenna *j* of the BS
$\gamma_{k}$	Signal-to-noise ratio of ED *k*
$\gamma_{\max}$	Maximum achievable signal-to-noise ratio
*B*	System bandwidth
$B_{k}$	Bandwidth allocated to ED *k* (equal to *B* in this work)
$R_{k}\left[t\right]$	Achievable data rate of ED *k* at time slot *t*
$m_{min}$	Minimum of the number of transmit and receive antennas
$\lambda_{l}$	*l*-th eigenvalue obtained from the SVD of $H_{k}$
$T_{k}^{l}$	Local computation latency of ED *k*
$T_{k}^{r}$	Remote computation latency of ED *k*
$T_{k}^{\mathrm{initialization}}$	Initialization latency including reattempts and waiting time
$T_{k}^{\mathrm{offloading}}$	Offloading transmission latency
$T_{k}^{\mathrm{computing}}$	Remote computation latency at the MEC server
$T_{S}$	Time duration of a successful initialization attempt
$T_{W}$	Waiting time before a reattempt
$T_{0}$	Time-slot duration for one transmission round
$n_{k}^{\mathrm{offloading}}$	Number of transmissions required for offloading by ED *k*
ϵ	Residual delay term for result return or control overhead
$L_{k}$	Task completion latency of ED *k*
$f_{k}$	Computing capability of ED *k*
$f_{s}$	Computing capability of the MEC server
$d_{k}$	Required CPU cycles per bit at ED *k*
$d_{s}$	Required CPU cycles per bit at the MEC server
$s_{k}^{t}$	State (observation) of agent *k* at time *t*
$a_{k}^{t}$	Action selected by agent *k* at time *t*
$r_{k}^{t}$	Reward of agent *k* at time *t*
$Q(s_{k},a_{k};\phi )$	Q-function parameterized by ϕ for agent *k*
$Q_{t}\left(s_{k},a_{k};\phi_{t}\right)$	Target Q-function
ε	Exploration rate
ζ	Discount factor
τ	Target network update factor
$K_{\max}$	Number of training episodes
$T_{\max}$	Maximum number of time steps per episode

**Table 3:** Simulation parameters.

Parameters	Values
Number of antennas at the BS (*J*)	4, 8, 16
Number of antennas at the ED (*I*)	1, 2
Number of edge devices (*K*)	1∼15
Maximum signal-to-noise ratio (${\mathrm{SNR}}_{max}$)	20 dB
System bandwidth (*B*)	10 MHz
Data Size ($\alpha_{k},\forall k$)	0.1 Gbits
Computing capability of device ($f_{k},\forall k$)	0.5, 1, 2 GHz/s
Computing capability of server ($f_{s}$)	100 GHz/s
Required CPU cycles ($d_{s},d_{k}$)	10 Hz
Waiting time before a reattempt ($T_{W}$)	60 ms
Time duration for reattempt success ($T_{S}$)	5 ms
DQN hidden layers	2 fully connected layers
Neurons per hidden layer	24
Activation function	ReLU
Output layer size	11 (equal to number of discrete offloading actions)
Target network update (smoothing) factor (τ)	0.5

## Data Availability

No new data were created or analyzed in this study.

## Abbreviations

The following abbreviations are used in this manuscript:

AI	Artificial Intelligence
ALOHA	Additive Links On-line Hawaii Area (protocol)
ARP	Access Reservation Protocol
BS	Base Station
CPU	Central Processing Unit
CSI	Channel State Information
CTDE	Centralized Training and Decentralized Execution
D2D	Device-to-Device
DNN	Deep Neural Network
DRL	Deep Reinforcement Learning
DQN	Deep Q-Network
ED	Edge Device
GHz	Gigahertz
GFMA	Grant-Free Multiple Access
Hz	Hertz
IoT	Internet of Things
MADQN	Multi-Agent Deep Q-Network
MADRL	Multi-Agent Deep Reinforcement Learning
MEC	Mobile Edge Computing
MIMO	Multiple-Input Multiple-Output
RL	Reinforcement Learning
SNR	Signal-to-Noise Ratio
UAV	Unmanned Aerial Vehicle
6G	Sixth-Generation Wireless Network
